# Molecular and functional crosstalk between extracellular Hsp90 and ephrin A1 signaling

**DOI:** 10.18632/oncotarget.22370

**Published:** 2017-11-03

**Authors:** Abdelkader Daoud, Udhayakumar Gopal, Jasmine Kaur, Jennifer S. Isaacs

**Affiliations:** ^1^ Department of Cell and Molecular Pharmacology, Medical University of South Carolina, SC, 29412, Charleston, USA; ^2^ Current address: Department of Pathology, Duke University School of Medicine, NC, 27708, Durham, USA

**Keywords:** extracellular Hsp90 (eHsp90), RhoA, myosin, Src, EphA2

## Abstract

The Eph receptor tyrosine kinase family member EphA2 plays a pivotal role in modulating cytoskeletal dynamics to control cancer cell motility and invasion. EphA2 is frequently upregulated in diverse solid tumors and has emerged as a viable druggable target. We previously reported that extracellular Hsp90 (eHsp90), a known pro-motility and invasive factor, collaborates with EphA2 to regulate tumor invasion in the absence of its cognate ephrin ligand. Here, we aimed to further define the molecular and functional relationship between EphA2 and eHsp90. Ligand dependent ephrin A1 signaling promotes RhoA activation and altered cell morphology to favor transient cell rounding, retraction, and diminished adhesion. Exposure of EphA2-expressing cancer cells to ligand herein revealed a unique role for eHsp90 as an effector of cytoskeletal remodeling. Notably, blockade of eHsp90 via either neutralizing antibodies or administration of cell-impermeable Hsp90-targeted small molecules significantly attenuated ligand dependent cell rounding in diverse tumor types. Although eHsp90 blockade did not appear to influence receptor internalization, downstream signaling events were augmented. In particular, eHsp90 activated a Src-RhoA axis to enhance ligand dependent cell rounding, retraction, and ECM detachment. Moreover, eHsp90 signaling via this axis stimulated activation of the myosin pathway, culminating in formation of an EphA2-myosin complex. Inhibition of either eHsp90 or Src was sufficient to impair ephrin A1 mediated Rho activation, activation of myosin intermediates, and EphA2-myosin complex formation. Collectively, our data support a paradigm whereby eHsp90 and EphA2 exhibit molecular crosstalk and functional cooperation within a ligand dependent context to orchestrate cytoskeletal events controlling cell morphology and attachment.

## INTRODUCTION

The family of Eph receptors and their cognate ephrin ligands play diverse physiological roles in development, axonal guidance, and vasculogenesis [1, 2]. Over the last decade, several Eph receptors have also emerged as key participants in cancer progression [1]. Among these, EphA2 has been validated as a predominant effector in a wide range of malignancies, including breast, prostate, GBM, and melanoma [2–8]. EphA2 is frequently overexpressed in solid tumors, an occurrence associated with metastatic potential and poor outcome [2, 7, 9, 10]. More recently, EphA2 has been shown to support drug resistance in a number of cancer models [11–16], further highlighting its multifaceted and direct role in tumor progression.

Eph receptors mediate cell-cell interactions and cell adhesion largely through their ability to signal via ligand dependent or independent modalities [17, 18]. While many of the invasive and metastatic properties of EphA2 are attributed to ligand-independent signaling [7, 15, 19, 20] ephrin A1-mediated ligand activation of EphA2 is generally antagonistic in action, leading to cell repulsion and tumor suppression [18, 19, 21–23]. EphA2 signaling can modulate the activity of Rho GTPases [24–26], which function as key regulators of adherens junction stability and cell shape [27, 28]. Ligand dependent signaling promotes RhoA activation and associated profound changes in cell morphology characterized by cell rounding and retraction, along with diminished cell-cell and cell-ECM interactions [26, 29, 30]. Hence, the contextual presence of ligand dramatically influences the ability of EphA2 to elicit cell adhesive or repulsive forces to impact the behavior of tumor cells.

EphA2 collaborates with a growing universe of signaling molecules [5, 13, 31–35]. Within this context, we previously reported that EphA2 exhibits functional collaboration with extracellular Hsp90 (eHsp90) [20]. Although Hsp90 has a well-established intracellular role in mediating the folding and activity of numerous clients, including EphA2 [36], cell surface and secreted forms of eHsp90 are frequently reported in tumor models [37, 38]. Increasing evidence points to a role for eHsp90 as a pro-motility and invasive factor in diverse cancers. Although the mechanistic basis of its tumor-promoting function is not well defined, eHsp90 dependent AKT activation is required for EphA2 invasive action within defined models [20]. These findings illustrate that eHsp90 and EphA2 may co-regulate cytoskeletal events to govern tumor cell movement.

In the current study, we aimed to further define the molecular and functional relationship between EphA2 and eHsp90 within the context of ligand activation. Towards this goal, we explored whether eHsp90 may influence ligand dependent EphA2 functions such as cell retraction and rounding. Our findings reveal that eHsp90 activation of a Src-RhoA axis enhances ligand dependent cell rounding and retraction. Notably, these effects were observed in diverse cancer models including breast, prostate, melanoma and GBM, indicating a conserved mode of action. Moreover, eHsp90 signaling via this axis stimulated activation of the myosin pathway, culminating in formation of an EphA2-myosin complex central for cytoskeletal remodeling. Collectively, our data support a paradigm whereby eHsp90 and EphA2 exhibit molecular crosstalk and functional cooperation within a ligand dependent context to orchestrate cytoskeletal events controlling cell morphology and attachment.

## RESULTS

### eHsp90 blockade impairs ephrin A1-dependent cell rounding

**Figure 1:**
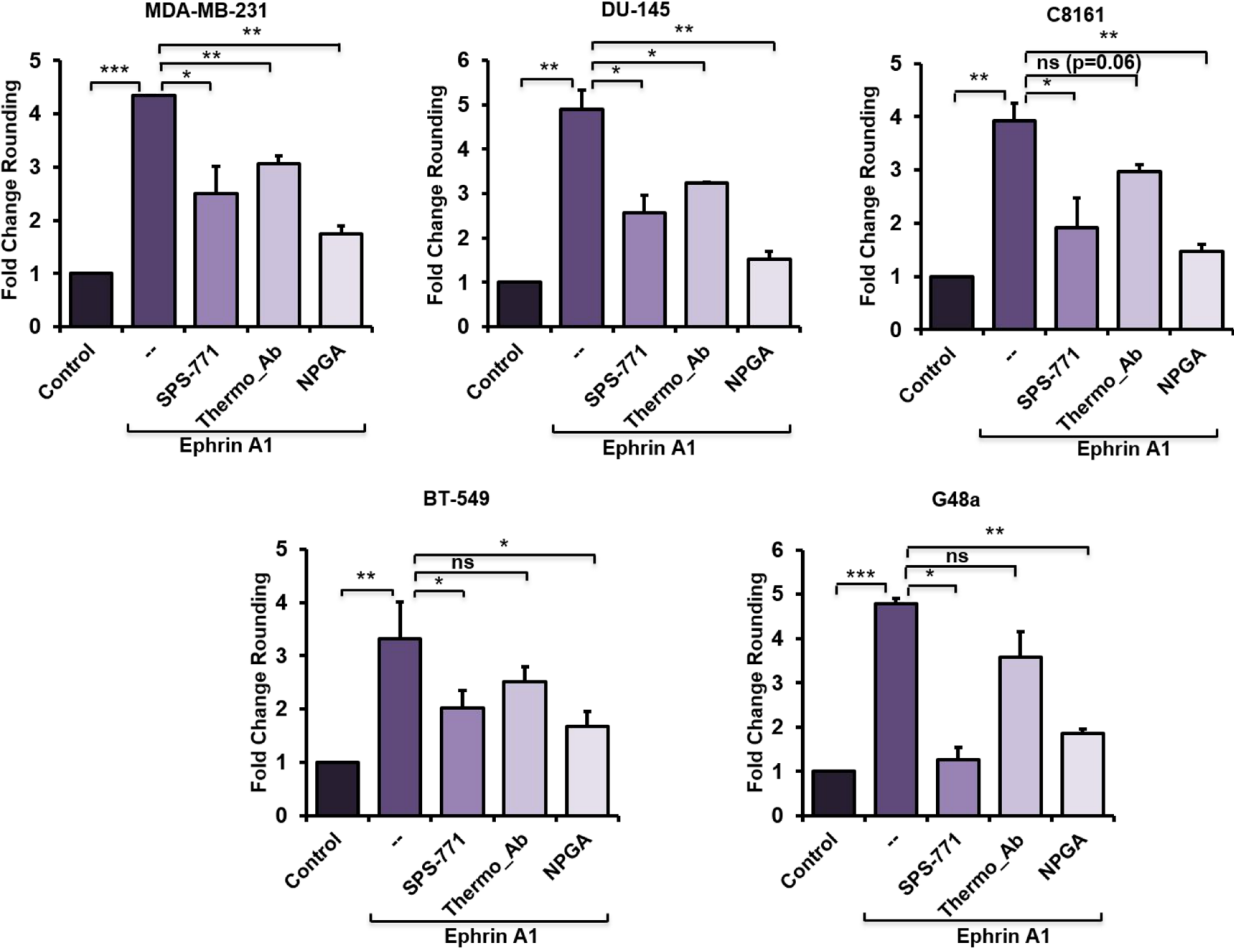
eHsp90 blockade impairs ephrin A1-dependent cell rounding The indicated cell types were pretreated 4 hr with either the Hsp90 blocking Ab SPS-771 (10 µg/ml) or NPGA (1.5 µM) followed by a 20 min incubation with ephrin A1 (1 µg/ml). Pictures were taken (20×) with a Nikon Eclipse TE2000-S and rounded cells were counted using ImageJ plugin CellCount as described in “Methods”. Data represents means from at least two biologically independent experiments. Statistical Analysis was performed using the Student’s *t*-test on GraphPad Prism. ^*^ = *p* < 0.05, ^**^p < 0.01, ^***^p < 0.001, ns = not significant.

It is well known that transient stimulation of EphA2-expressing cells with a soluble recombinant ephrin A1 fused to the Fc portion of IgG (hereafter referred to as ephrin A1), promotes rapid cell rounding, contractility and repulsion [26, 29]. Therefore, a ligand-initiated cell morphology assessment was performed in a panel of EphA2-expressing cancer cell lines to assess the potential functional involvement of eHsp90. We, and others, have shown that eHsp90 action can be neutralized by exposure of intact cells to a subset of Hsp90-specific antibodies [20, 37, 39–41]. Hence, representative tumor cells from a number of models were subjected to a 4 hour exposure to two different Hsp90 antibodies with known epitopes localized to the N-terminal portion of Hsp90. The rationale for this approach was based upon the finding that this domain of Hsp90 recapitulates many of the pro-motility effects of eHsp90 [42], coupled with our prior demonstration that the Hsp90 targeting antibody SPS-771 inhibits cancer cell motility [20, 37]. As a complementary treatment, cells were incubated with the cell-impermeant small molecule geldanamycin derivative (NPGA), shown to be specific for eHsp90 [43], without interfering with the functions of intracellular Hsp90. Following these eHsp90-targeted neutralizing treatments, cells were then transiently stimulated with ephrin A1 ligand. As shown (Figure 1, Supplementary Figure 1A), the SPS-771 Hsp90 Ab and NPGA effectively diminished ligand dependent cell rounding in each evaluated cell line, with NPGA demonstrating the largest suppressive effect. Interestingly, the Thermo antibody was effective in 2 of 5 cell lines, indicating that tumor cells may exhibit variable Hsp90 epitopes on the cell surface, or that accessory proteins may hinder recognition. Interestingly, differential epitope display has been demonstrated for EphA2 [44]. Nonetheless, these findings indicate that eHsp90 cooperates with ephrin A1 to enhance cell rounding.

Recent reports have indicated the possibility that eHsp90 may shuttle from the extracellular space to an intracellular location [45]. We therefore evaluated whether the utilized antibodies may be targeting an intracellular chaperone population. MDA-MB-231, shown to express eHsp90 [39, 41], were incubated with fluorescently labeled PE-conjugated Hsp90 Ab in either permeabilized or unpermeabilized cells. Laminin was used as a membrane marker to facilitate cell detection. Findings from these experiments indicated that Hsp90 Ab was predominantly found at the cell surface (Supplementary Figure 2A). To further substantiate these findings, and to confirm that fluorescent labeling did not modify functional properties, we compared FITC-conjugated GA to NPGA. It was previously shown that FITC-GA renders the drug cell-impermeant [43, 46], and functionally comparable to NPGA. As shown (Supplementary Figure 1C, 1D), both GA-FITC and NPGA similarly impaired ephrin A1 dependent cell rounding. Although conceivable that eHsp90 participates in a shuttling mechanism, our findings indicate that within the evaluated timeframe, a predominantly extracellular form of Hsp90 facilitates the observed ligand dependent morphological changes.

### Blockade of surface Hsp90 does not alter ephrin-induced EphA2 internalization

**Figure 2:**
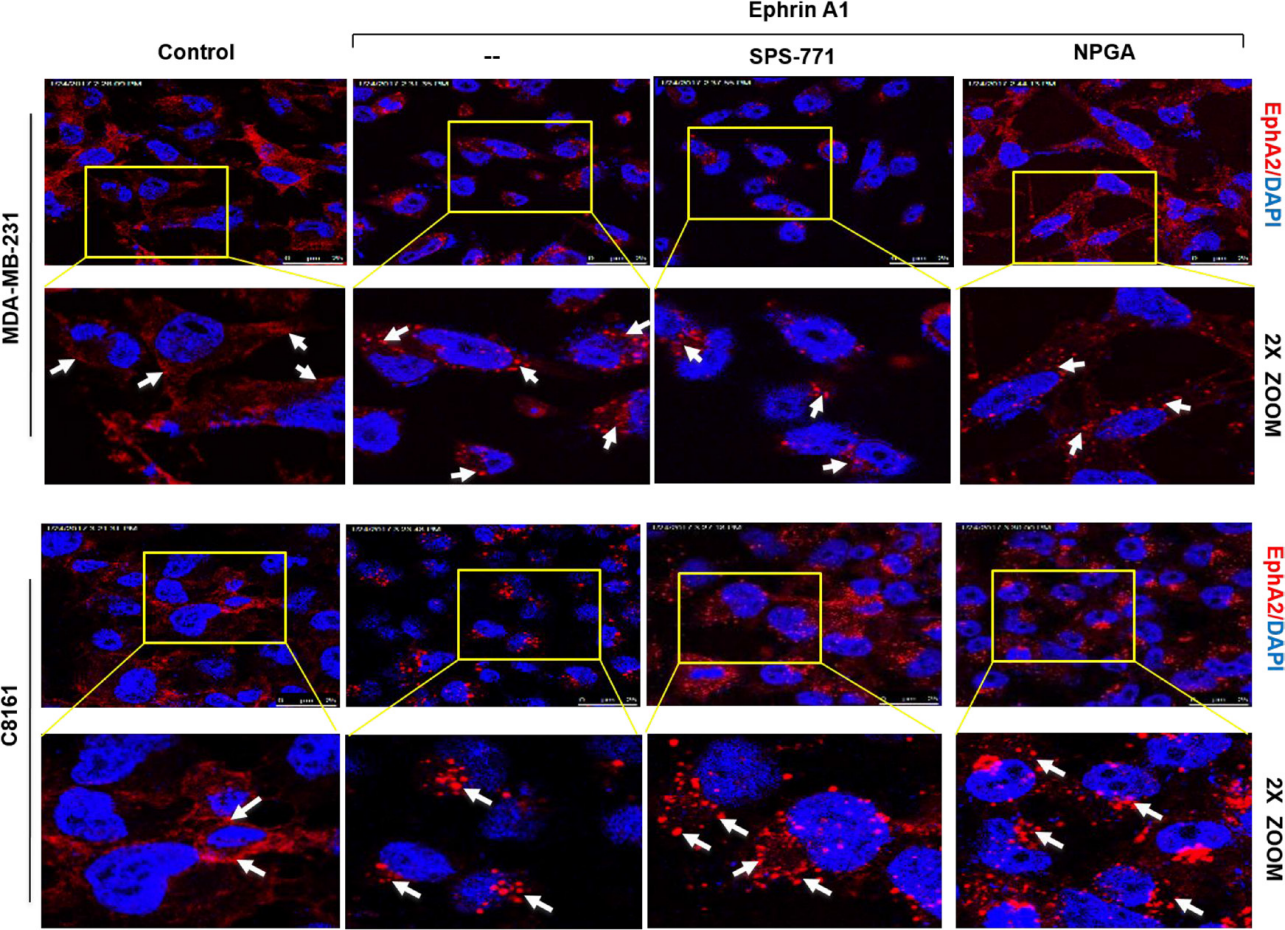
Blockade of surface Hsp90 does not alter ephrin-induced EphA2 internalization MDA-MB-231 and C8161 cells were pretreated with either SPS-771 or NPGA for 4 hr followed by ephrin A1 treatment. Immunofluorescence images were taken on a Leica SP5 confocal microscope at 63x. Scale bar = 25 µm (Top panel of each cell line).

It is well established that ligand stimulation of tumor cells promotes EphA2 receptor internalization [47]. Given that eHsp90 blockade impaired cell rounding, we next explored whether eHsp90 neutralization impacted receptor internalization. MDA-MB-231 breast and C8161 melanoma cells were pretreated with either SPS-771 or NPGA, followed by ligand stimulation. As shown (Figure 2), in all instances, the EphA2 receptor rapidly internalized and demonstrated a typical vesicular punctate pattern. This trend was also observed in additional cancer cell models, such as in DU145 prostate and U373 GBM cells (Supplementary Figure 2). These data indicate that eHsp90 blockade impacts cell rounding in a pathway that appears uncoupled from EphA2 receptor internalization.

### An eHsp90-Src signaling axis regulates ephrin-dependent cell rounding and adhesion

**Figure 3:**
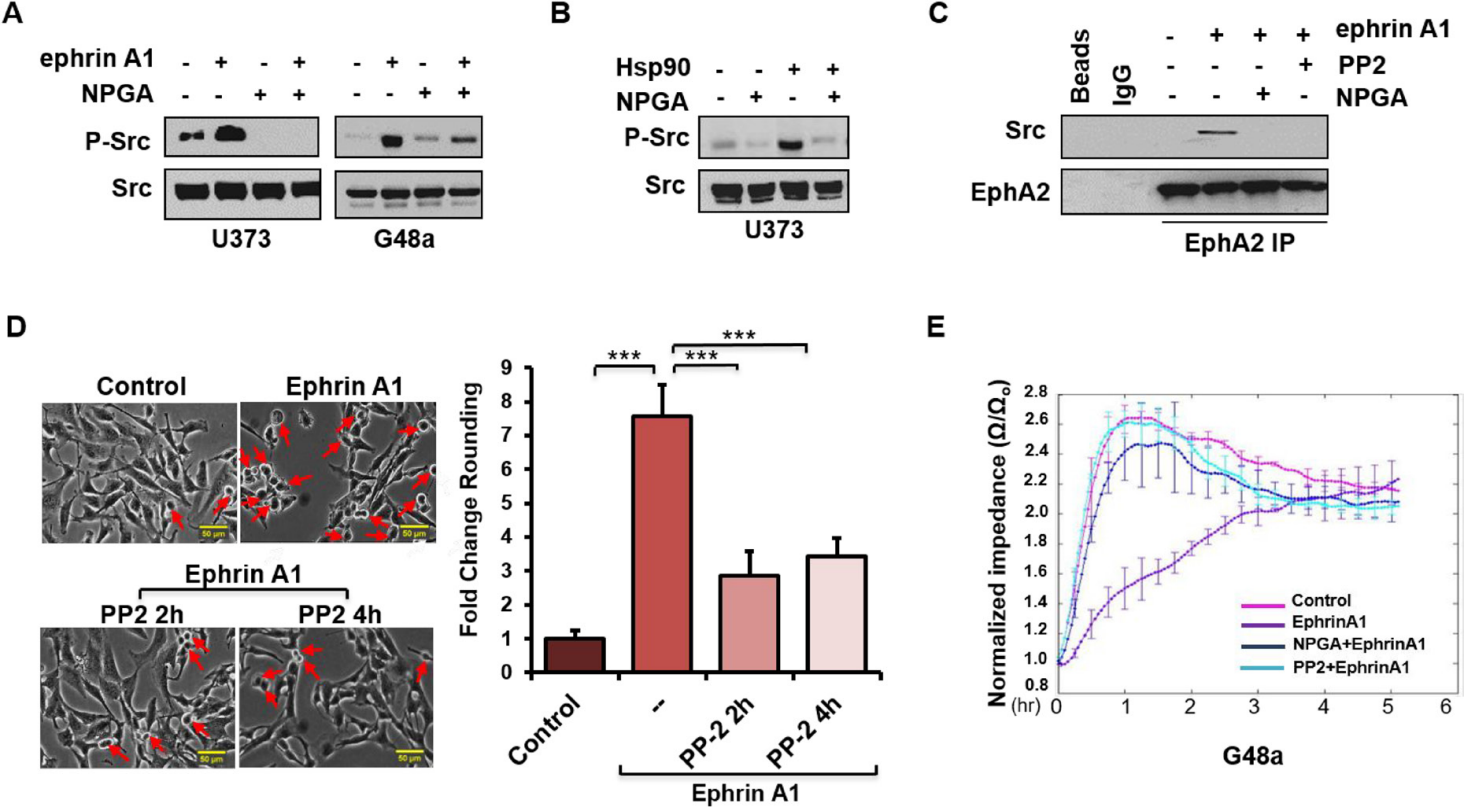
An eHsp90-Src signaling axis regulates ephrin-dependent cell rounding and adhesion (**A**) Cells were subjected to an 8 hr pretreatment with NPGA, as indicated, followed by a 20 min. exposure to ephrin A1. Resultant lysates were immunoblotted for P-Src416. (**B**) U373 cells were serum starved for 8 hr and stimulated with Hsp90a protein (3 µg/ml) for 15 min. Resultant lysates were evaluated for P-Src416. (**C**) U373 cells were pretreated (16 hr) with either the src inhibitor PP2 (20 µM) or NPGA, followed by transient ephrin A1 stimulation. The interaction between EphA2 and Src was assessed from EphA2 immunoprecipitates (IPs). In parallel, control IPs were performed, as indicated, with protein A sepharose beads alone (Beads), or with beads coupled to an isotype-matched IgG control antibody (IgG). (**D**) G48a cells were treated with PP2 for either 2 or 4 hr, as indicated, followed by transient ephrin A1 stimulation. Pictures were taken (20×) with a Nikon Eclipse TE2000-S. Rounded cells are indicated by red arrows. The fold change in cell rounding was calculated as described from duplicate experiments. Scale bar = 50 µm. (**E**) ECIS evaluation of cell attachment of U373 cells pretreated with either NPGA or PP2, followed by immediate stimulation with ephrin A1. Results are representative of data from three independent experiments.

We next sought to understand the molecular events involved in eHsp90-dependent regulation of ephrin signaling. Although a number of effectors have been implicated in this pathway, we initially focused on Src and Rho, given their significant roles in EphA2 signaling and ligand dependent cytoskeletal remodeling [26, 30, 48–51]. As shown (Figure 3A), ephrin A1 stimulated Src phosphorylation in the indicated glioma cell lines, congruous with prior reports documenting src activation in response to ligand [26]. Interestingly, NPGA robustly attenuated ephrin-dependent Src activation, supporting the notion of functional cooperativity between eHsp90 and Src. In further support of this crosstalk, U373 glioma cells demonstrated robust Src activation upon treatment with exogenous Hsp90 protein, the specificity of which was confirmed by abrogation by NPGA (Figure 3B). We next evaluated whether eHsp90 action influenced the interaction between EphA2 and Src. While ephrin A1 facilitated the association between EphA2 and Src, this interaction was lost following blockade of either eHsp90 or Src (Figure 3C). As Src is implicated in ligand-mediated contractility [26] we confirmed that blockade of Src signaling with PP2 significantly reduced ephrin A1 induced cell rounding in G48a cells (Figure 3D) and other cell types (not shown).

ephrin A1 cell rounding correlates with transiently diminished cell-ECM attachments. This weakening of substratum attachments, mediated in part by transient inhibition of integrin activity [29, 52] facilitates cell repulsion and contraction. To quantitatively assess the effects of Src upon ephrin mediated cellular attachment, U373 cells were pretreated with either PP2 or NPGA followed by transient ligand addition, and impedance, a measure of cell attachment, was analyzed via the ECIS assay [53, 54]. As shown (Figure 3E), whereas untreated cells demonstrated a robust increase in impedance within an hour of plating, indicative of cell attachment and spreading, ephrin A1 treatment significantly reduced this impedance by over 2.5. Consistent with our molecular data, pretreatment of cells with either PP2 or NPGA completely antagonized ligand mediated impedance loss. These findings further support the premise that an eHsp90-Src pathway plays a central role in ephrin A1 dependent cell detachment and repulsion.

### Impairment of Rho signaling phenocopies the morphological effects of eHsp90 blockade in response to ligand

**Figure 4:**
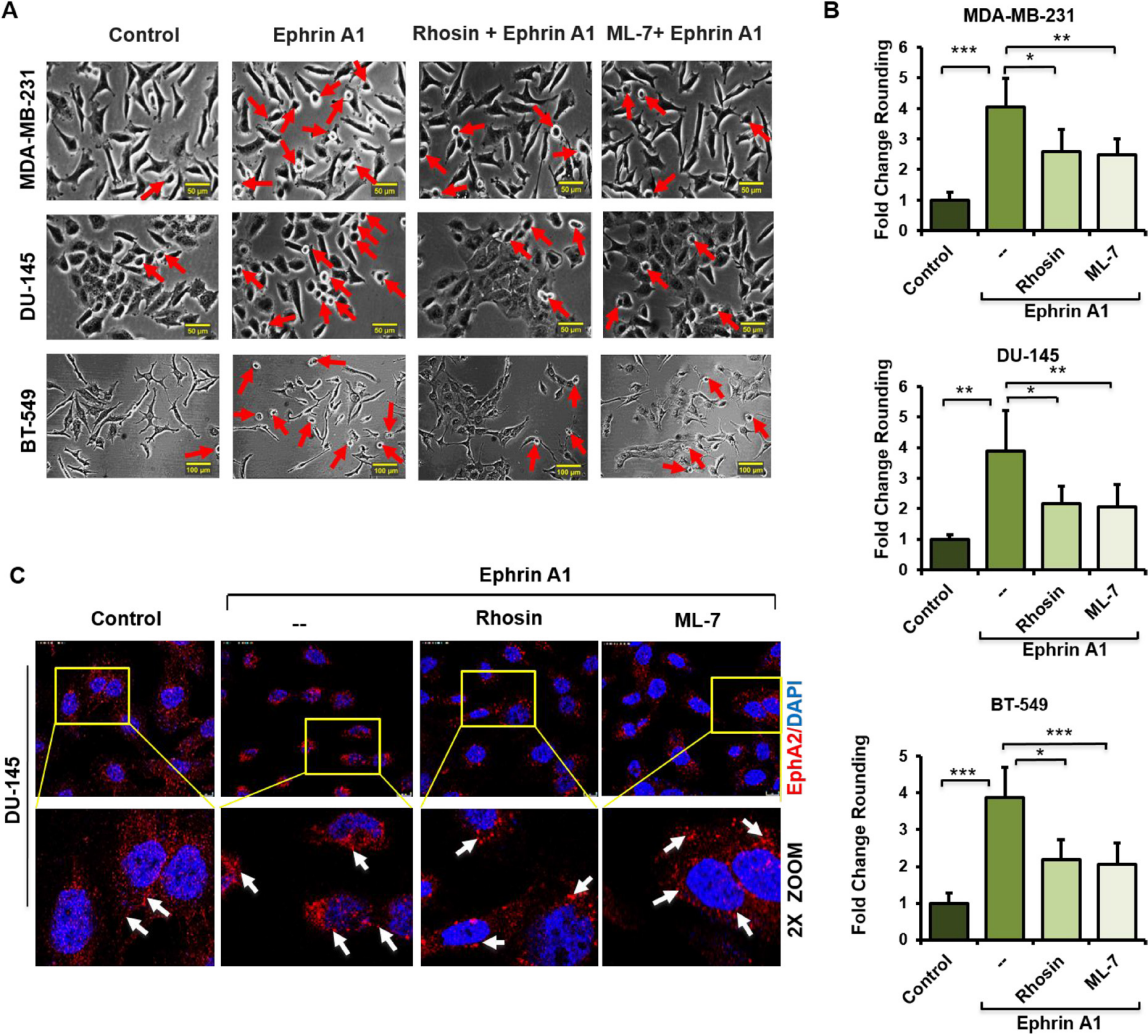
Impairment of Rho signaling phenocopies the morphological effects of eHsp90 blockade in response to ligand (**A**, **B**) The indicated cell types were pretreated 4 hr with the Rho inhibitors Rhosin (30 µM) or the MLCK inhibitor ML-7 (10 µM) prior to ephrin A1 treatment. Pictures were taken with a Nikon Eclipse TE2000-S and the fold change of rounded cells was calculated as described. Rounded cells are indicated by red arrows. Scale bar = 50 µm. (**C**) Cells were treated as above and EphA2 immunofluorescence images were taken 20 min following ephrin A1 treatment on a Leica SP5 confocal microscope at 63×. Data represent the means from at least two independent experiments. Statistical Analysis was performed using the Student’s *t*-test on GraphPad Prism. ^*^ = *p* < 0.05, ^**^p < 0.01, ^***^p < 0.001, ns = not significant. Scale bar = 10 µm (Top panel).

Prior reports have established that Rho signaling plays a conserved role in ephrin A1 dependent cytoskeletal rearrangements and cell rounding [26, 49]. We confirmed these findings, evidenced by the ability of the Rho pathway inhibitors Rhosin, a Rho GEF binding domain inhibitor, and ML7, a selective inhibitor of myosin light chain kinase, to significantly attenuate the morphological effects of ligand in a variety of cell types. Representative data for breast and prostate lines are shown (Figure 4A, 4B). Conversely, treatment of breast or glioma cells with the Rho activator CN03A, which blocks GTPase activity leading to constitutively active Rho, enhanced ligand dependent cell rounding (Supplementary Figure 3). These findings highlight the essential role of Rho in directing ligand-dependent morphological changes. Interestingly, and similar to our findings with NPGA treatment, Rho pathway inhibition did not appreciably diminish ligand-mediated EphA2 internalization (Figure 4C), indicating that receptor internalization and cell rounding are likely uncoupled events.

### Extracellular Hsp90 stimulates a Src-Rho pathway critical for myosin-dependent EphA2 activity

**Figure 5:**
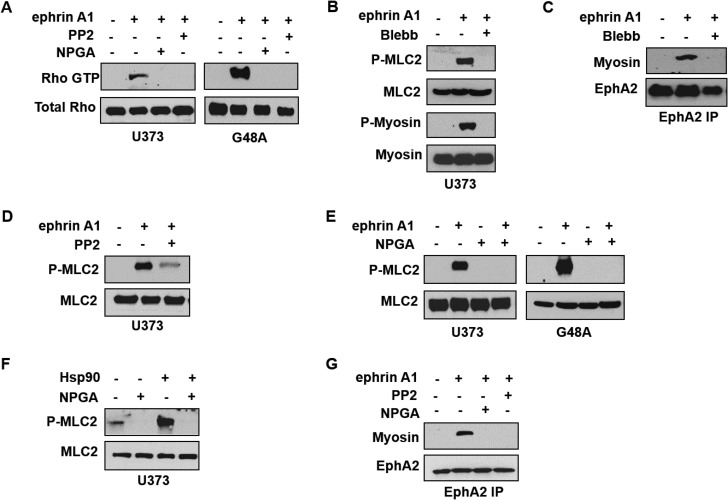
Extracellular Hsp90 stimulates a Src-Rho pathway critical for myosin-dependent EphA2 activity (**A**) The indicated cell types were pretreated (16 hr) with either PP2 or NPGA followed by transient ephrin A1 stimulation, and active Rho was detected from resultant lysates by immunoprecipitation of GST- tagged Rhotekin protein-binding domain, as described. (**B**) U373 cells were stimulated with ephrin A1 in the absence or presence of pretreatment (16 hr) with the myosin inhibitor blebbistatin (10 µM), and resultant lysates were probed for indicators of myosin activity (P-myosin S1943, P-MLC2 Thr18/Ser19). (**C**) U373 cells were treated as in B, and EphA2 was immunopreciptated from 1 mg of the indicated lysates, followed by immunoblot analysis of EphA2 and co-precipitating myosin. (**D**) Myosin activation was assessed from U373 cells pretreated with Src inhibitor (16 hr PP2 pretreatment), followed by transient ephrin A1 stimulation. (**E**) U373 and G48a cells were pretreated with NPGA, as indicated, prior to ephrin A1 ligand addition, and resultant lysates probed for total and P-MLC2. (**F**) The effect of Hsp90a protein (15 min) upon myosin activation was assessed in starved (8 hr) U373 cells. (**G**) The effects of Src inhibition and eHsp90 blockade (16 hr) upon EphA2-myosin interaction was assessed in U373 cells as in C.

Given that Rho serves as a downstream effector of Src signaling [55], an occurrence also reported in response to ephrin A1 stimulation [26], we next evaluated whether the newly identified eHsp90-Src pathway was capable of regulating Rho activity. Using 2 glioma cell lines as representative model systems, we validated the ability of ligand to stimulate Rho GTPase activity via a GST-Rhotekin pull-down assay (Figure 5A). Consistent with our morphological data, blockade of either eHsp90 or Src abrogated Rho activation. Rho effectors stimulate myosin-driven contractility by promoting the phosphorylation of myosin light chain (MLC) on serine 19, which promotes the assembly of myosin into filaments that stabilize actin-myosin interactions [56, 57]. We therefore evaluated the phosphorylation status of myosin light chain 2 (MLC2) as a surrogate for Rho activity. As shown (Figure 5B), ligand stimulates the phosphorylation of both MLC2 as well as the downstream substrate myosin II (P-myosin), an effect that is abrogated by the myosin pathway inhibitor blebbistatin [58]. Similar molecular results were obtained with use of specific Rho inhibitors (data not shown).

Although EphA2 and myosin functionally cooperate to elicit ligand-dependent cell rounding, few studies have evaluated the direct interaction between EphA2 and myosin [59]. We show that ligand promotes the interaction of EphA2 receptor with myosin, an effect that is abrogated by blebbistatin (Figure 5C). Having substantiated that ligand phosphorylates MLC2 and promotes EphA2-myosin interaction, we next explored the potential effect of the eHsp90-Src axis upon this pathway. As shown (Figure 5D, 5E), blockade of either Src or eHsp90 effectively prevented MLC2 phosphorylation in response to ligand. To further validate a direct role for eHsp90, U373 cells were treated with exogenous eHsp90 protein, which robustly stimulated MLC2 phosphorylation (Figure 5F). Consistent with these findings, we further demonstrated that blockade of either Src or eHsp90 similarly prevented ligand dependent EphA2-myosin association (Figure 5G). Collectively, these data indicate that an eHsp90-Src pathway plays a significant role in RhoA-myosin activation and subsequent EphA2 directed cellular contractility.

## DISCUSSION

Extracellular Hsp90 is emerging as a conserved facilitator of cell motility and invasion [37]. The increased detection of eHsp90 expression in malignancy indicates that tumor cells may be more reliant upon eHsp90 for their pro-invasive behavior. Despite this increasingly accepted function, a molecular framework for eHsp90 action is lacking. A central feature of cell migration is the extensive reorganization of the actin cytoskeleton. The Rho family of small GTPases are key regulators of actin cytoskeletal reorganization [60], and represent a point of convergence between cell motility and morphology. Our current study positions eHsp90 as an effector of RhoA activation and as a facilitator of ligand mediated EphA2 signaling and subsequent cell contractility. To our knowledge, this is the first report to demonstrate molecular and functional crosstalk between eHsp90 and Ephrin signaling.

**Figure 6:**
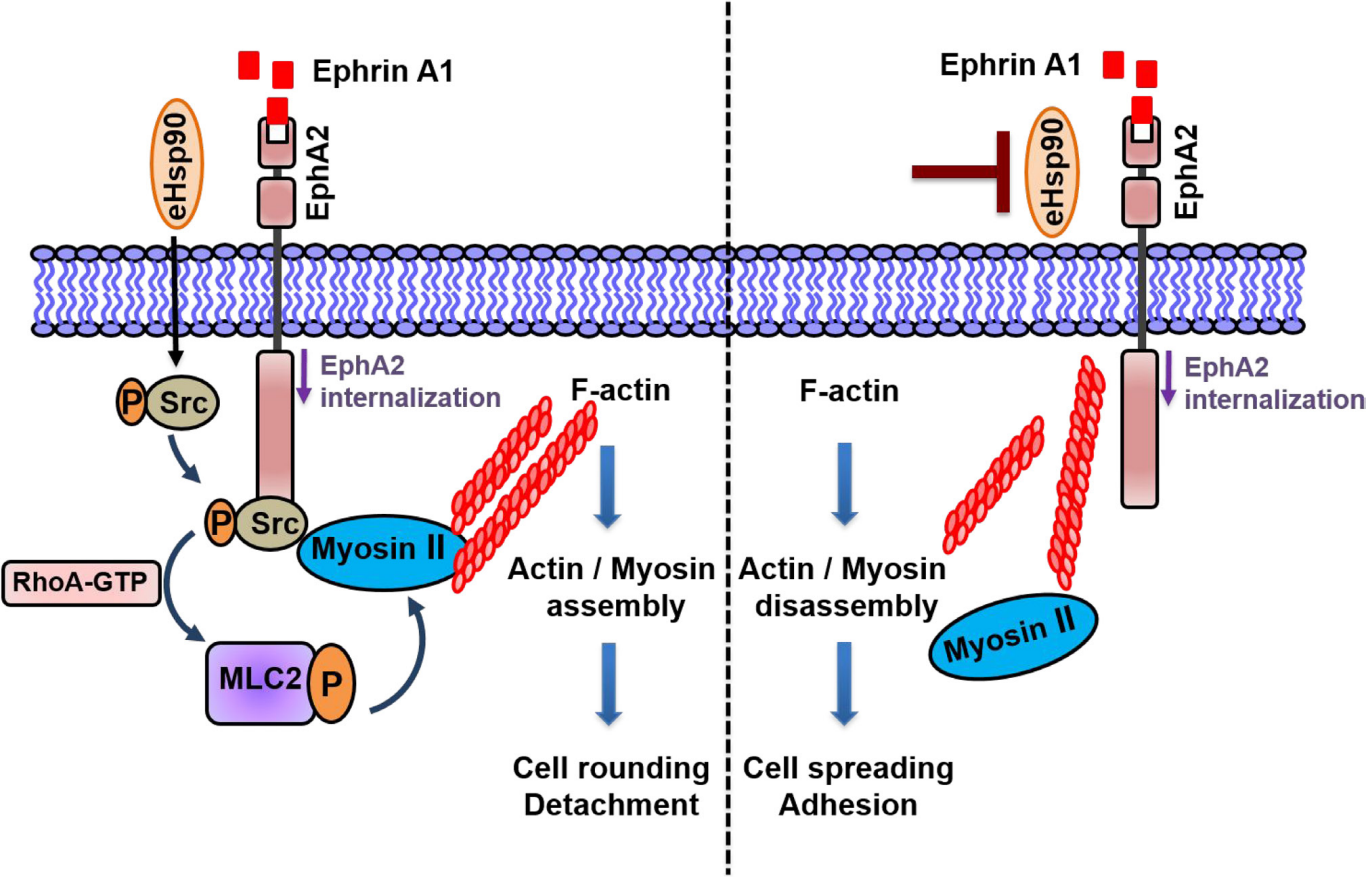
Model for eHsp90 dependent crosstalk with ephrinA1 dependent signaling The presence of eHsp90 (left side), initiates signaling events that stimulate Src activation, leading to downstream molecular events such as RhoA activation, MLC2 phosphorylation, and assembly of EphA2-myosin complexes. In the context of eHsp90 and ephrin A1 ligand, these molecular events promote acto-myosin assembly and subsequent cell rounding, repulsion and cell-ECM detachment. The targeting of eHsp90 (right side), attenuates the Src-RhoA axis, preventing EphA2-myosin assembly, thereby interfering with ligand dependent cell rounding. In this scenario, cells remain morphologically flat and spread due to ECM adhesive forces. EphA2 internalization is not appreciably affected by eHsp90.

Initial evidence of this novel crosstalk was provided by morphological data demonstrating that a relatively short term (4 hr) blockade of eHsp90 was sufficient to significantly diminish ephrin A1 dependent cell rounding. Notably, these results were obtained following eHsp90 targeting with two distinct Hsp90 antibodies, or with cell-impermeable NPGA. Moreover, these trends were consistent among diverse cancer cell models, implicating a conserved mode of action. Interestingly, eHsp90 blockade did not prevent ligand mediated receptor internalization, indicating that its effects primarily impacted downstream signaling events subsequent to ligand engagement. Upon further investigation, we found that eHsp90 elicited Src activation and, conversely, that eHsp90 blockade impaired ephrin A1-mediated Src activation and formation of an EphA2-Src complex (Figure 6). Functionally, this eHsp90-Src axis cooperated to initiate both ligand dependent cell rounding and detachment. On a molecular level, this signaling axis was required for RhoGTPase activity, myosin activation, and formation of an EphA2-myosin complex. Collectively, our data support the premise that eHsp90 signaling has the capacity to augment both the molecular and functional effects of ephrin ligand. These findings considerably build upon the existing framework for eHsp90 action in malignancy and highlight its previously unappreciated ability to modulate cytoskeletal dynamics converging upon cell morphology, motility and invasion.

Eph receptors and their ephrin ligands mediate intercellular communication by interacting at contact sites. Although ephrin A1 is a preferred ligand for EphA2, ephrin A1 may communicate with other EphA receptors [61–63]. Although we cannot discount the possibility that additional Eph receptors may be involved, our findings clearly demonstrate a requirement for eHsp90 in facilitating productive EphA2 protein complexes with Src and myosin. While eHsp90 activated both Src and myosin, eHsp90 stimulation alone was insufficient to elicit cell rounding in the absence of ligand (data not shown). Thus, while eHsp90 co-stimulates ligand dependent signaling, it is unable to fully phenocopy the morphological effects of ligand.

Future studies are warranted to determine whether eHsp90 is a bona fide ligand for EphA2, or whether it may serve as an accessory protein with other ligands. The recent discovery of progranulin as a novel EphA2 ligand [64] provides support for the notion that disparate proteins may recognize EphA2 as a cognate receptor within a cancer context. It is also conceivable that eHsp90 cooperates with additional proteins to mediate Src-Rho signaling and/or cell retraction. For instance, eHsp90 may signal via the promiscuous LRP1 receptor [65], a protein also known for regulating cell motility and adhesion [66]. Although the molecular intricacies of potential accessory proteins remain to be clarified, our current findings highlight that myriad signaling pathways have the capacity to augment ephrin signaling.

We previously reported the ability of eHsp90 to co-regulate EphA2 invasive action in a ligand independent manner via an AKT pathway [20]. Ligand engagement suppresses AKT activation and changes the molecular output to favor cell retraction [18]. Our current findings reveal that eHsp90 plays a dual role in modulating ligand-dependent and –independent EphA2 signaling and function. This revelation provides new insights into eHsp90 action and further implicates eHsp90 as an effector of cytoskeletal remodeling and morphological plasticity. This notion that eHsp90 may function as a rheostat of cytoskeletal dynamics is also fully consistent with our prior findings that eHsp90 regulates epithelial to mesenchymal transitions [37]. Interestingly, EphA2 overexpression is also correlated with amoeboid invasion [67, 68], whereby tumor cells exhibit enhanced morphological plasticity to enable cellular deformation and passage through spatially restrictive spaces [69–71]. Given that EphA2 promotes amoeboid invasiveness in a ligand independent manner [67], it is conceivable that ancillary factors such as eHsp90 may cooperate with EphA2 to enhance the RhoA activation associated with this motility style [72]. Hence, our current study has revealed new molecular and functional aspects of eHsp90 action within the context of EphA2 signaling and beyond.

## MATERIALS AND METHODS

### Reagents

Recombinant Mouse Ephrin-A1/FC Chimera, (602-A1-200, lot AOK1716012) and Recombinant Human IgG1-Fc (110-HG-100, lot EAX0616051) were purchased from R&D systems. Mouse anti-ECK/EphA2, clone D7 antibody (05-480, lot 1993900) was obtained from Millipore. Rabbit Hso90α polyclonal antibodies (ADI-SPS-771-J, lot 02011345) and the PE conjugate (ADI-SPS-771PE-200, lot 08191023) were purchased from Enzo Life Sciences. Rabbit Hsp90 alpha polyclonal antibody (PA3-013, lot RE235232) was from ThermoFisher Scientific. Laminin antibody was from Abcam (ab11575, lot GR46031-9). Antibodies to P-MLC2 (Thr18/Ser19) (3674S, lot 5), MLC2 (8505S, lot 4), P- Myosin IIa (Ser 1943) (5026S, lot 1), Myosin IIa (3403S, lot 1), Phospho-EphA2 (Tyr772) (8244S, lot 1) were purchased from Cell Signaling Technologies. The Alexa fluor conjugated secondary IgG antibodies goat anti-rabbit 633 (A21070, lot 45419A), goat anti-rabbit 488 (A11008, lot 1797971), and goat anti-mouse 633 (A21050, lot 690316) were purchased from ThermoFisher Scientific. MLCK inhibitor ML-7 (475880), Src inhibitor PP2 (529573) and the Rho inhibitor Rhosin (555460, lot 2825564) were from Calbiochem Millipore. Rho activator II (CN03) was from Cytoskeleton. The small molecule cell-impermeant geldanamycin (NPGA), also known as DMAG-N-oxide modified geldanamycin [43] was synthesized by Chris Lindsey and Craig Beeson (Drug Discovery, Medical University of South Carolina) as previously reported [73].

### Cell culture

The glioma cell line U373 was obtained from Dr. Frank Furnari (University of California, San Diego) while G48a, provided by Dr. Waldemar Debinski (Wake Forest School of Medicine, NC) was derived from a high grade glioma [74]. Human breast cancer cell lines MDA-MB-231 (ATCC) and BT-549 and human prostate cancer DU-145 was obtained from ATCC. Human melanoma C8161 cells [75] were provided by Dr. Mary Hendrix (Northwestern University, Chicago). U-373 were cultured in Eagle’s Minimum Essential Medium (EMEM) (Corning, 10-009-CV, lot 22416005) containing 1.5 g/L sodium bicarbonate, L-glutamine, non-essential amino acids (NEAA), Sodium Pyruvate and 10% fetal bovine serum (FBS) (Gibco, 10437-028, lot 1778586). All other cell lines were cultured in RPMI medium (Hyclone SH30096.02, lot AB10179406) supplemented with 2 mM L-glutamine (Hyclone, SH30034.02, lot AXH43923), 1% NEAA (Gibco, 11140-050, lot 04018), 1 mM sodium pyruvate (Hyclone, SH30239.01, lot AAL207014), 10 mM HEPES (Hyclone, SH30237.01, lot AZM197541) and 10% FBS. All cells were maintained at 37°C in a humidified incubator with 5% CO2.

### Western blot

Cell extracts for Western blot analysis were prepared and performed as described [20, 37]). Briefly, blots were incubated overnight at 4°C with the indicated primary antibody diluted in 5% milk and subsequently developed with SuperSignal West Pico Chemiluminescent reagents (Thermo Scientific, 34080). All immunoblots are representative of a minimum of two independent experiments.

### Cell rounding assessment

Cells were plated at 5 × 10^4^ cells/ml in 6-well plates and allowed to adhere for 48 hours. At 70% of confluence, cells were pretreated with the indicated agents followed by a 20 min stimulation with 1ug/ml ephrin A1-Fc. Treatment with a non-specific IgG1-Fc confirmed the specificity of ligand-dependent cell rounding.

Representative images were captured from 6 different fields for each condition using a Nikon Eclipse TE2000-S inverted microscope with a 20× objective. Rounded cells were counted from each picture using the ImageJ plugin CellCount. Typically, between 20–50 cells were captured in each field and the percentage of rounded/total were calculated and normalized to the control untreated group to calculate the fold change. Each experiment was performed in duplicate.

### Immunofluorescence

Cells were grown on 18 mm coverslips (VWR, 48380-046) within 12-well plates and allowed to adhere for 48 hr. Coverslips were washed twice with PBS and then fixed with 4% paraformaldehyde (Thermo Scientific, 28908, lot RK2302081) for 15 min at room temperature; cells were then washed trice with PBS containing 10mM of glycine and either permeabilized with 0.1% Triton-X 100 (Calbiochem, 9410, lot UB16DZEMS) in PBS for 5 min, or left intact. Cells were subsequently washed three times with PBS and blocked in 3% milk for 1 hour at room temperature. Cells were incubated with primary antibodies (1:100 in blocking buffer) overnight at 4°C and washed twice prior to incubation with secondary antibodies (1:200 in blocking buffer) for 1 hour at room temperature. Cells were then washed twice and mounted with ProLong Gold Antifade Mount with DAPI (Thermo Scientific, P36931, lot 1836709). Images were taken on a Leica SP5 confocal microscope with a 63× glycerol immersion lens.

### Rho activity assay

Cellular assessment of Rho activation was determined via Rhotekin pull-down assays, as described in the Rho activation Assay Kit (16116, ThermoFisher Scientific). Briefly, after the indicated cell treatments, cells were washed, lysed, and clarified cell lysate was incubated with GST-Rhotekin coupled beads for 30 min. Bound proteins were eluted in SDS-PAGE sample buffer and subjected to immunoblot analysis.

### Electrical Cell substrate Impedance Sensing (ECIS)

Quantitative evaluation of cell adhesion was measured by the electrical impedance assay (ECIS), as described [54]. Briefly, U373 cells were pretreated with NPGA and PP2 for 16 hrs. Cells were subsequently plated (1.5 × 10^5^ cells/well) onto 8W10E PET ECIS arrays (Ibidi, 72010) precoated with 100 μg/ml human plasma fibronectin (Life Technologies, 33016-015) in 0.15 M NaCl, 0.01 M Tris, pH 8.0 buffer. ephrin A1 was added to the indicated wells immediately prior to data acquisition and impedance levels were measured in real time with an Applied Biophysics ECIS 1600 instrument. Graphs were generated with Prism GraphPad.

## SUPPLEMENTARY MATERIALS FIGURES



## Abbreviations

AKT: Protein kinase B (PKB)
ECIS: Electric Cell-substrate Impedance Sensing
eHsp90: Extracellular heat shock protein 90
EphA2: Ephrin type A receptor 2
ECM: Extracellular Matrix
ERK: Extracellular signal–regulated kinase
GEFs: Guanine-Exchange Factors
GBM: Glioblastoma multiforme
GST: Glutathione S-transferase
GTP: Guanosine triphosphate
LRP-1: LDL Receptor-like protein 1
MLC2: Myosin light chain 2 or Regulator light chain 2 (RLC2)
MLCK: Myosin light chain kinase
NPGA: Non-permeable geldanamycin
RhoA: Ras homolog gene family member A
Src: Cellular Src kinase
