# Supplementary material for: Molecular and functional crosstalk between extracellular Hsp90 and ephrin A1 signaling
Source: Oncotarget. 2017 Nov 3;8(63):106807–19. doi: 10.18632/oncotarget.22370 (PMC5739775; doi:10.18632/oncotarget.22370)
Supplement: Supplementary file 1 [file oncotarget-08-106807-s001.pdf]

Molecular and functional crosstalk between extracellular Hsp90 and ephrinA1 signaling

SUPPLEMENTARY MATERIALS

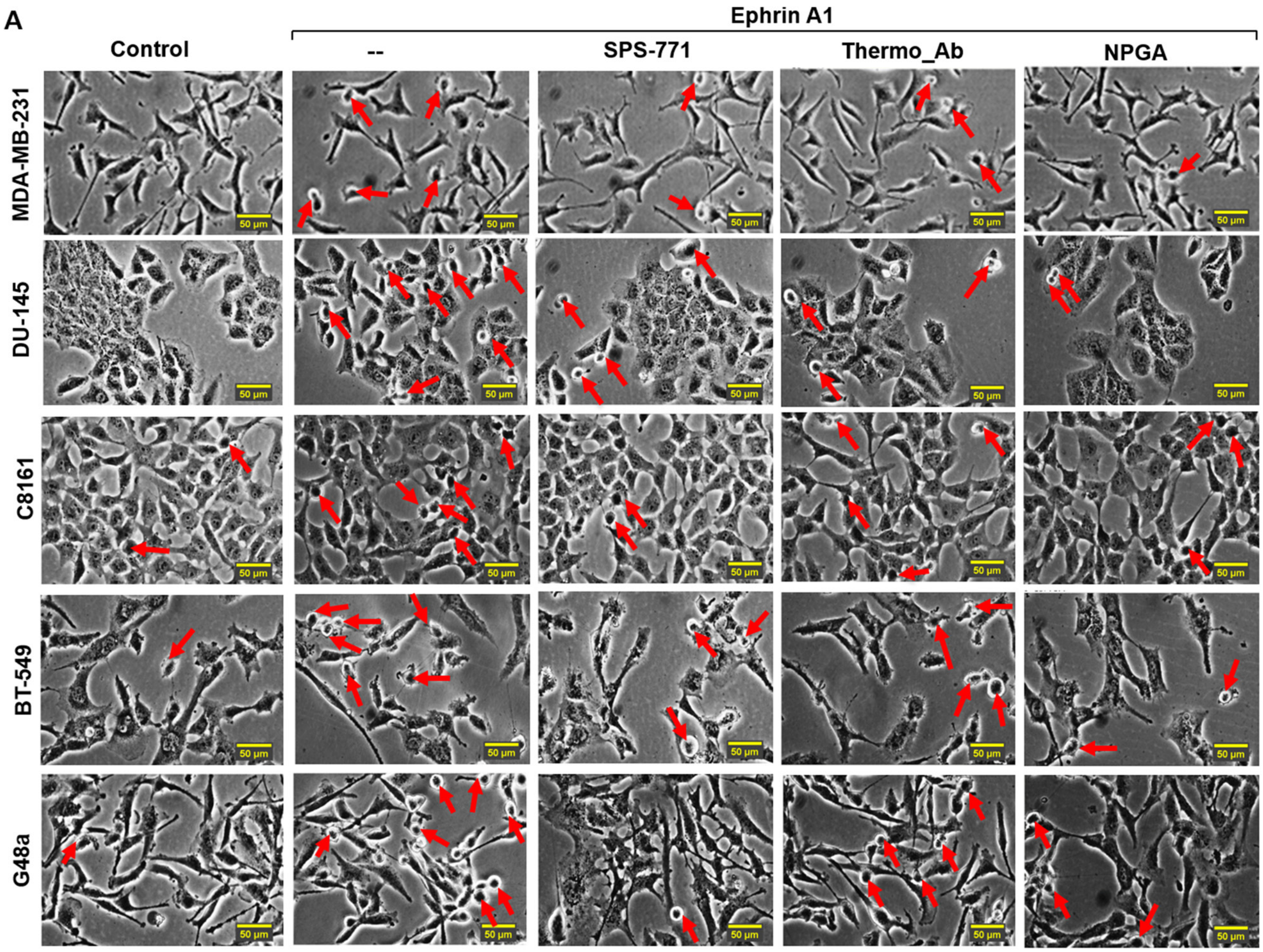

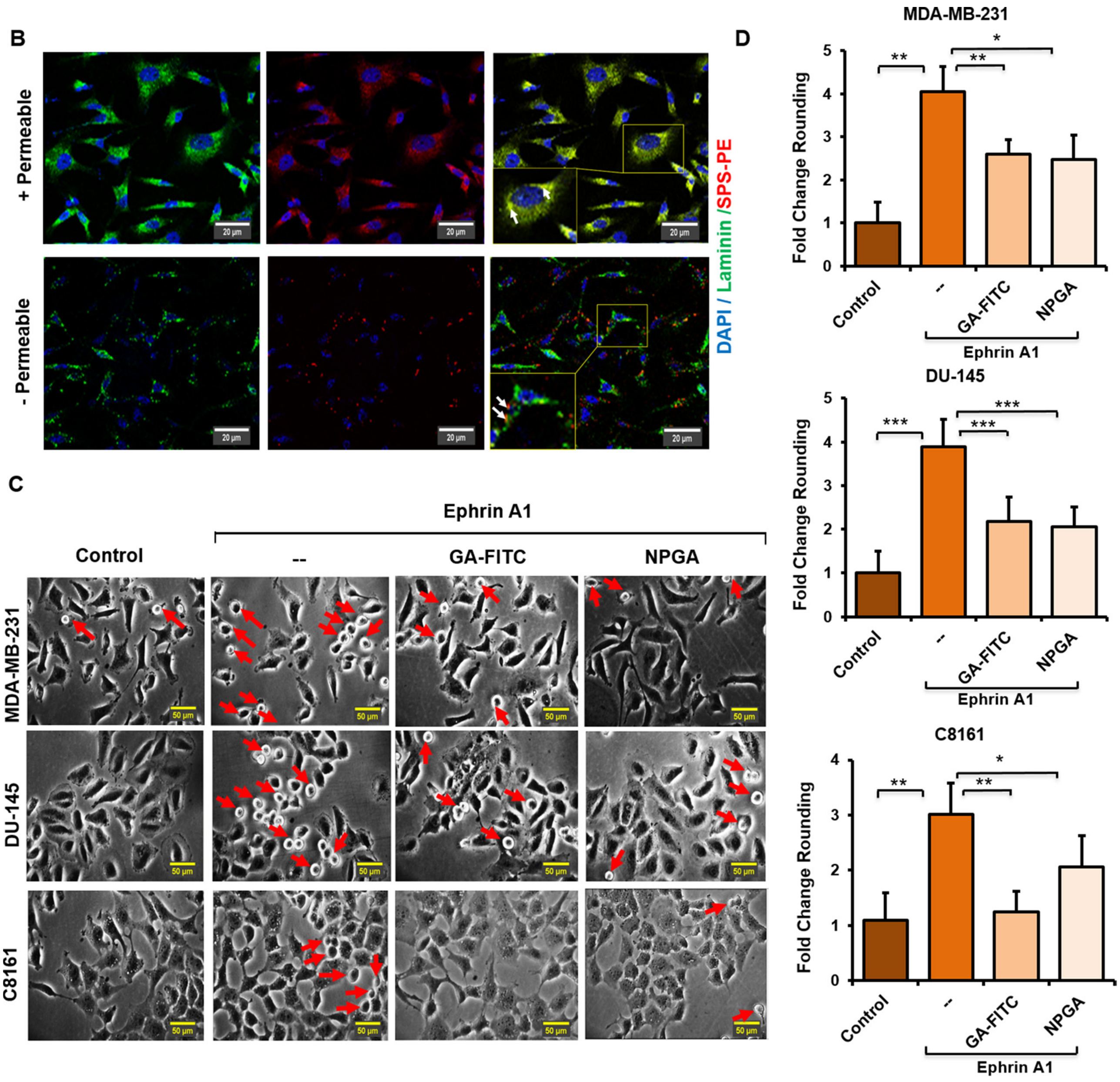

Supplementary Figure 1: Ability of eHsp90-targeted agents to impair ephrin A1-dependent cell rounding. (**A**) The indicated cell types were were pretreated (4 hr) with SPS-771 (10 µg/ml), Thermo Ab (10 µg/ml), or with NPGA (1.5 µM) followed by transient (20 min) stimulation with Ephrin-A1 ligand (1µg/ml). G48a were pretreated for 16 hours before ligand addition. Representative images were taken (20×) with a Nikon Eclipse TE2000-S. Rounded cells are indicated by red arrows. Scale bar = 50 µm. (**B**) MDA-MB-231 cells were either pretreated (4 hr) with PE-labeled Hsp90 Ab (SPS-771-PE, 10 ug/ml) and then fixed (No permeabilization) or fixed and permeabilized and subsequently incubated with SPS-771-PE (Permeabilization). Laminin was used as a basal membrane marker. Immunofluorescence was performed as described in "Methods". Images were taken on a Leica SP5 confocal microscope at 63×. Surface Hsp90 is indicated by white arrows. Scale bar = 20 µm. (**C**, **D**) The indicated cell types were treated (4 hr) with either FITC-coupled GA (GA-FITC) or with NPGA at 1.5µM for 4 hours prior to Ephrin-A1 stimulation. Pictures were taken under a Nikon Eclipse TE2000-S and rounded cells were counted using ImageJ plugin CellCount as described in "Methods". Rounded cells are indicated by red arrows. Data of Rounded cells represents means from at least two independent experiments. Statistical Analysis was performed using the Student's *t*-test on GraphPad Prism. $^{*} = p < 0.05$, $^{**}p < 0.01$, $^{***}p < 0.001$, ns = not significant. Scale bar = 50 µm.

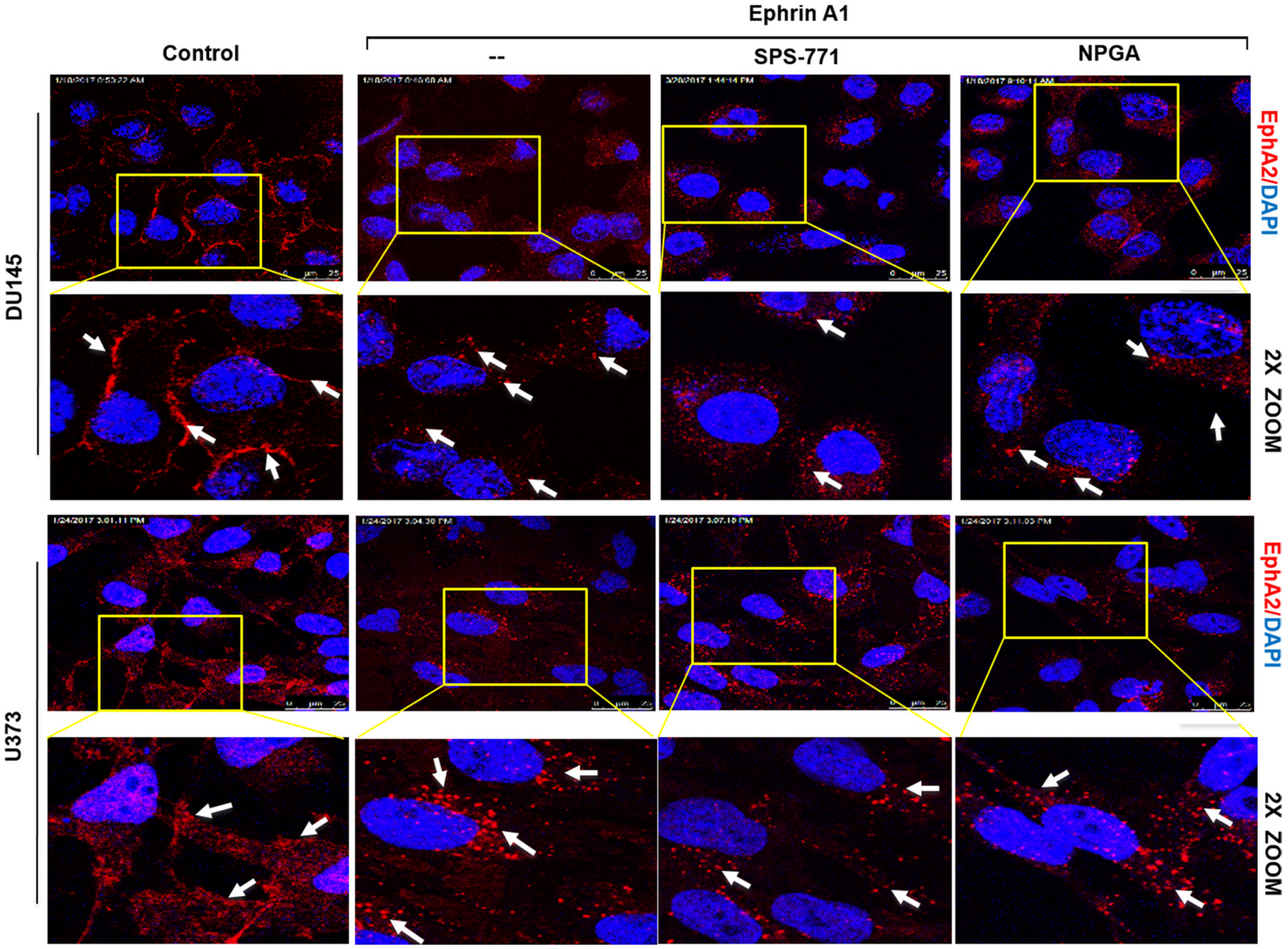

Supplementary Figure 2: Blockade of eHsp90 and subsequent impairment of ephrin mediated cell rounding does not alter EphA2 internalization. DU-145 and U-373 cells were pretreated with SPS-771 or NPGA (4 hr) prior to ligand stimulation. Immunofluorescence for EphA2 was performed as described. Images were taken on a Leica SP5 confocal microscope at 63×. Scale bar = 25um (Top panel of each cell line).

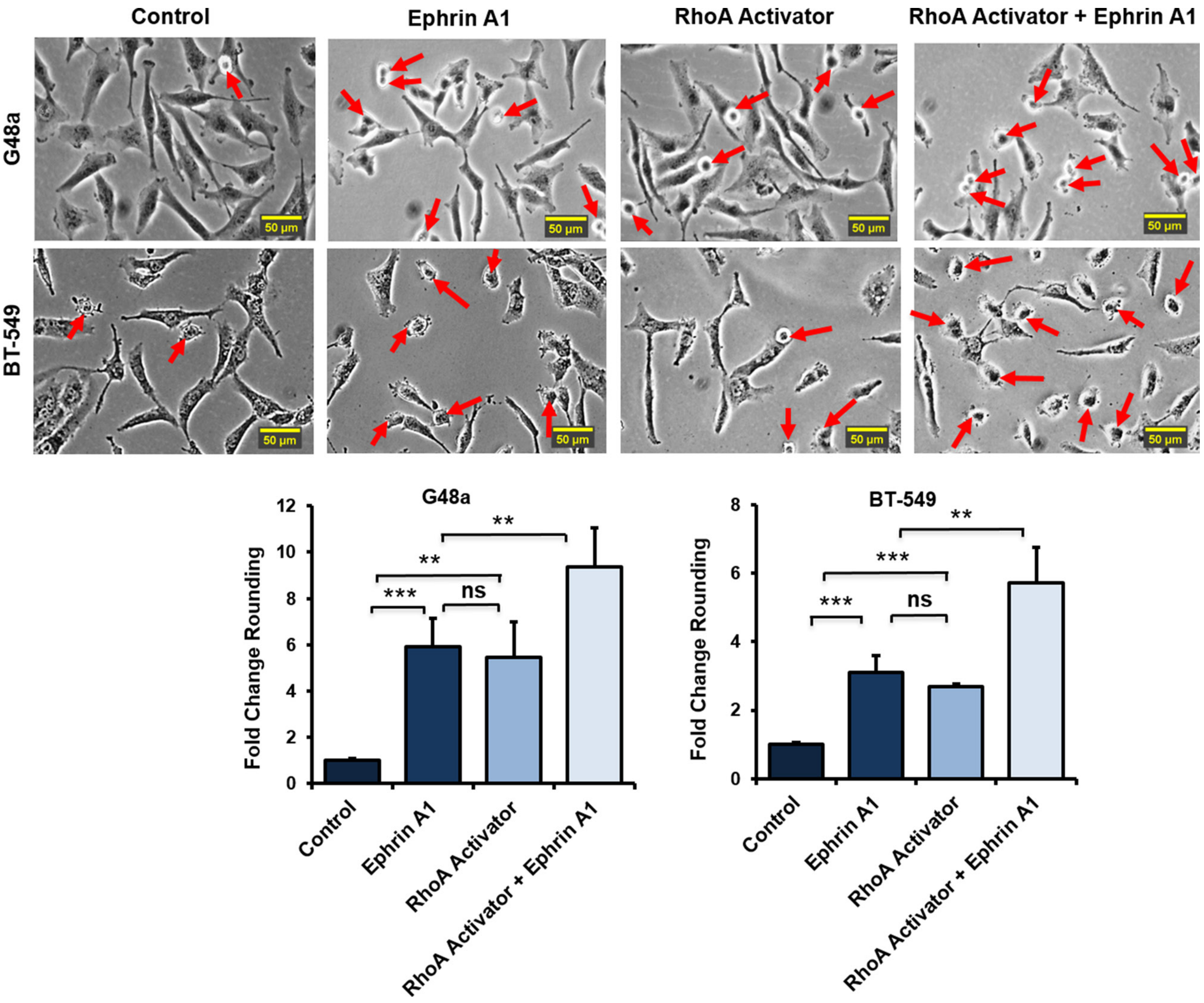

Supplementary Figure 3: RhoA activator enhances Ephrin-A1 induced cell rounding. G48a and BT-549 cells were treated (4 hr) with RhoA activator II (Cytoskeleton, CN03) at 2 µg/ml followed by transient Ephrin A1 stimulation. Images were acquired (20×) with a Nikon Eclipse TE2000-S and rounded cells were counted using the ImageJ plugin CellCount as described. Rounded cells are indicated by red arrows. Data of Rounded cells represents means from at least two independent experiments. Statistical Analysis was performed using the Student's *t*-test on GraphPad Prism. $^{*} = p < 0.05$, $^{**}p < 0.01$, $^{***}p < 0.001$, ns = not significant. Scale bar = 50 µm.
